# Urgent Implantation of Peritoneal Dialysis Catheter in Chronic Kidney Disease and Acute Kidney Injury—A Review

**DOI:** 10.3390/jcm12155079

**Published:** 2023-08-02

**Authors:** Hanna Cholerzyńska, Wiktoria Zasada, Hanna Michalak, Miłosz Miedziaszczyk, Andrzej Oko, Ilona Idasiak-Piechocka

**Affiliations:** Department of Nephrology, Transplantology and Internal Medicine, Poznan University of Medical Sciences, 61-701 Poznan, Poland; hanna.cholerzynska@gmail.com (H.C.); wiktoriazasada8@gmail.com (W.Z.); hania.michalak1998@gmail.com (H.M.); aoko@ump.edu.pl (A.O.); ilonaidasiak@poczta.onet.pl (I.I.-P.)

**Keywords:** peritoneal dialysis, urgent-start peritoneal dialysis, chronic kidney disease, acute kidney injury

## Abstract

Acute kidney injury (AKI) and sudden exacerbation of chronic kidney disease (CKD) frequently necessitate urgent kidney replacement therapy (UKRT). Peritoneal dialysis (PD) is recognized as a viable modality for managing such patients. Urgent-start peritoneal dialysis (USPD) may be associated with an increased number of complications and is rarely utilized. This review examines recent literature investigating the clinical outcomes of USPD in CKD and AKI. Relevant research was identified through searches of the MEDLINE (PubMed), Scopus, Web of Science, and Google Scholar databases using MeSH terms and relevant keywords. Included studies focused on the emergency use of peritoneal dialysis in CKD or AKI and reported treatment outcomes. While no official recommendations exist for catheter implantation in USPD, the impact of the technique itself on outcomes was found to be less significant compared with the post-implantation factors. USPD represents a safe and effective treatment modality for AKI, although complications such as catheter malfunctions, leakage, and peritonitis were observed. Furthermore, USPD demonstrated efficacy in managing CKD, although it was associated with a higher incidence of complications compared to conventional-start peritoneal dialysis. Despite its cost-effectiveness, PD requires greater technical expertise from medical professionals. Close supervision and pre-planning for catheter insertion are essential for CKD patients. Whenever feasible, an urgent start should be avoided. Nevertheless, in emergency scenarios, USPD does remain a safe and efficient approach.

## 1. Introduction

Dialysis is a recognized kidney replacement therapy (KRT) for individuals afflicted with chronic kidney disease (CKD) or acute kidney injury (AKI). According to data from the US Renal Data System (USRDS) in 2020, the prevalence of end-stage renal disease (ESRD) reached 807,920 cases, with 480,516 patients undergoing in-center hemodialysis (HD), 65,406 receiving peritoneal dialysis (PD), 11,916 opting for home hemodialysis, and 245,846 undergoing transplantation [1]. Similar figures were observed in Poland, where the number of CKD patients was estimated at 4.2 million, with 18,847 individuals receiving hemodialysis, 800 undergoing peritoneal dialysis, and 748 receiving transplantation in 2020 [2,3,4]. In recent years, there has been a notable surge in ESRD incidence, consequently increasing the demand for suitable and effective KRT options [1]. While the decision regarding the most appropriate kidney replacement therapy ideally involves collaboration between physicians and patients, with the establishment of a comprehensive schedule and plan, research conducted by Marrón et al. indicates that only 23% of patients received optimal care [5]. Additionally, 57.8% of the dialysis processes are initiated as unplanned, primarily due to factors such as non-compliance with follow-up appointments, as well as an unexpected rapid deterioration in the glomerular filtration rate (GFR) [5].

Hemodialysis remains the predominant modality of dialysis treatment, with peritoneal dialysis accounting for approximately 8% of all dialysis cases [5]. Multiple studies and reports suggest that PD is more cost-effective and better tolerated by patients compared to hemodialysis [1,6,7]. However, not all patients follow the preferred pathway of early detection, gradual decline in estimated GFR (eGFR), and planned initiation of dialysis. A French study reported that 30% of patients required an emergency dialysis start, defined as the initiation of dialysis within the first 24 h following a nephrology appointment, due to a life-threatening event [8]. The initiation of peritoneal dialysis necessitates the implantation of a PD catheter, typically followed by a two-week period of rest and care to allow the cuff to form a seal against leaks and to promote proper healing [9]. Thus, any dialysis initiated within the first two weeks after catheter implantation is classified as urgent-start peritoneal dialysis (USPD) [9,10,11]. Recently, the application of USPD has become more widespread worldwide [12], and it has been demonstrated to be as safe and effective as urgent-start hemodialysis (USHD) [9,10]. 

Acute kidney injury, which can result from sepsis, contrast-associated complications, or other urgent conditions, often requires prompt management, including KRT [13,14]. The selection of the most suitable approach remains a subject of ongoing investigation, where continuous renal replacement therapies (CRRT) and intermittent hemodialysis (IHD) constitute the most commonly employed methods [15]. However, as of late, a shift toward the utilization of peritoneal dialysis has been observed [16].

The objective of this review was to collect and analyze the potential complications and risk factors associated with the urgent implantation of a peritoneal catheter in cases of sudden exacerbation of chronic kidney disease and acute kidney injury, as presented in published studies. By examining the available evidence, we aim to gain insights into the outcomes of urgent-start peritoneal dialysis and identify areas for further research and improvement.

## 2. Materials and Methods

A comprehensive search of electronic databases, including MEDLINE (PubMed), Scopus, Web of Science, and Google Scholar, was conducted to collect relevant information regarding the implementation methods of dialysis catheters for planned/urgent PD and available therapeutic options for patients with AKI and CKD. The search terms used were carefully selected to ensure the retrieval of studies that align with the objectives of this review. Only studies published in English that outlined and reported treatment outcomes satisfied the inclusion criteria. Papers that failed to report the abovementioned were excluded from the review, ensuring the selection of high-quality and relevant literature for the present study.

## 3. Results

### 3.1. Technique and Preparation for USPD

#### 3.1.1. Preoperative Measures for USPD

Achieving optimal outcomes in peritoneal dialysis, whether planned or urgent, relies on the proper functioning of the catheter. In addition to discussing the treatment plan with the patient, physicians should thoroughly assess any potential factors that could complicate the procedure, such as hernias or past abdominal surgeries. It is also crucial to ensure that anticoagulant medications are not being used. Careful administration of the treatment and meticulous patient preparation contribute to favorable outcomes [9].

Determining the best exit site location requires consideration of various factors, including the patient’s belt line, skin creases and folds, placement of existing scars, chronic skin conditions, possible physical limitations, urinary incontinence, and even the patient’s bathing practices [17]. Pre-placement assessment using ultrasound plays a crucial role in accurately accessing the peritoneum, identifying and avoiding blood vessels and detecting any anatomical abnormalities [18] Ash et al. extensively described the invaluable assistance provided by ultrasonography during catheter placement [19]. However, it is important to note that fluoroscopy has also demonstrated efficacy in specific scenarios [19]. Fluoroscopy aids in verifying the accurate positioning of the needle, guidewire, and catheter during the procedure. It enables real-time visualization of the contrast medium as it expands within the peritoneum, confirming proper needle entry into the abdominal cavity. Furthermore, fluoroscopy helps track the needle’s trajectory through the rectus sheath and peritoneum, thereby enhancing the precision of the placement process. Additionally, it facilitates the identification of potential complications, such as bowel perforation, allowing for prompt intervention if necessary [19].

#### 3.1.2. Insertion and Selection of Dialysis Catheter for USPD

There is a range of available options with regard to catheter placement solutions. Physicians may choose percutaneous insertion of a peritoneal dialysis catheter (which is characterized by lower rates of infections and catheter migration), such as a modified Seldinger technique, laparoscopy (particularly useful in avoiding omental entrapment, due to the visualization of the entire omentum), as well as a peritoneoscopic procedure (which involves insertion of a rigid endoscope into the peritoneal space, its inspection, and direction of the catheter), or open dissection [20,21,22,23,24,25]. The safest puncture site should be determined by grayscale and Doppler ultrasonography and/or fluoroscopy [18,19]. The alternative placement techniques and catheter options include the Moncrief–Popovich technique (with a subcutaneously buried PD catheter), extended dialysis catheters (allowing placement of the exit site in remote locations), or self-locating catheters [26,27].

Catheter outcomes (including infectious or mechanical complications, functional parameters, and catheter survival) are similar between surgical and non-surgical insertion techniques. Therefore, patients without previous major abdominal surgeries are suitable to receive any one of the aforementioned methods [28].

The International Society for Peritoneal Dialysis (ISPD) guidelines recommend the use of silicone rubber catheters equipped with double Dacron cuffs [29]. An accepted standard for PD is the application of a straight/coiled-tip Tenckhoff catheter, with or without a preformed arc bend (with no significant difference shown in functionality) [9]. Apart from the choice of the catheter, determining the catheter insertion site, tunnel configuration, and exit site location are crucial for every successful PD. 

To date, no evidence-based recommendations have been established regarding the preferred catheter design or optimal insertion technique for USPD [12]. Operators should carefully select and implement the appropriate catheter length to avoid potential drain pain resulting from the irritation of the visceral structures (parietal peritoneum). It often results from placing the catheter too deep in the pelvis, particularly in the case of hydraulic suction and compression of the catheter side holes by nearby structures, thus leading to flow obstruction [17,29,30]. As a result, resorting to gravity-only drainage and performing the insertion of the paramedian catheter through the body of the rectus muscle may be effective in preventing such complications [17]. The choice of the PD catheter insertion technique does not significantly affect the initiation of USPD in ESRD patients [31]. 

**Figure 1:**
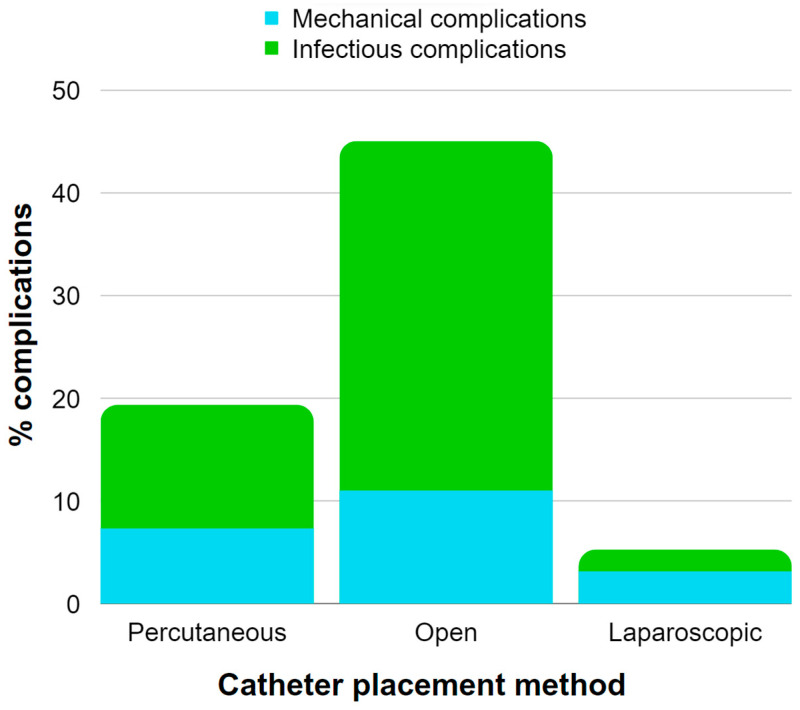
Illustration of the occurrence rates of mechanical and infectious complications associated with various catheter placement methods in the adult population with CKD and AKI undergoing USPD. The chart is constructed using data extracted from Table 1 [37,44,46].

Table 1 provides an overview of the catheter placement methods in the reviewed studies concerning USPD and the prevalence of complications. It is important to note that not all papers were included in the presented analysis due to missing data, and the second column highlights papers describing AKI in children. Figure 1 presents data extracted from Table 1, illustrating the occurrence rates of mechanical and infectious complications associated with various catheter placement methods in the adult population with CKD and AKI undergoing USPD. The most common catheter placement method in AKI was percutaneous catheter placement. Only one study reported using open surgical and laparoscopic methods in AKI, as the laparoscopic method requires more time to prepare, which makes it less convenient in urgent settings [32]. The associated complications were classified into three categories: (1) infectious, including peritonitis and catheter exit-site infections; (2) mechanical, such as catheter malfunction, obstruction, leakage, and poor flow; and (3) other associated conditions, e.g., hypotension, bleeding, and electrolyte instabilities. The most common complications involved mechanical ones, followed by infectious complications.

**Table 1:** Comparison of catheter placement methods in urgent-start PD in CKD and AKI.

Paper	AKI/CKD	Catheter Placement Method	Mechanical Complications	Infectious Complications	Other Complications
I. Kaplan Bulut (2016) [33]	AKI (in children)	percutaneous	catheter malfunction (19.6%)	peritonitis (6%), catheter exit-site infection (6%)	no other complications reported
P. Choudhary (2021) [34]	AKI (in children)	percutaneous	hemorrhagic effluent and obstruction in flow (6%), pericatheter leakage (4%)	peritonitis (4%)	no other complications reported
P. Coccia (2021) [32]	AKI (in children)	open (84%) laparoscopic (7.6%) percutaneous (8.4%)	catheter malfunction (24%), fluid leakage (11.5%)	peritonitis (19%),	bleeding events (6%), hyperglycemia (2%)
A. Al-Hwiesh (2018) [35]	AKI	percutaneous	no mechanical complications reported	Infections (9.5%)	hypotension (15.9%), bleeding events (6.3%), arrhythmias (7.9%), hypoglycemia (4.8%), hypomagnesemia (11.1%), hypocalcemia (9.5%), hypophosphatemia (11.1%), thrombocytopenia (4.8%)
D. Gabriel (2009) [36]	AKI	percutaneous	no mechanical complications reported	peritonitis (18%)	no other complications reported
D. Ponce (2012) [37]	AKI	percutaneous	mechanical complications (7.3%)	peritonitis (12%)	no other complications reported
D. Ponce (2013) [38]	AKI	percutaneous	no mechanical complications reported	peritonitis (16.3%)	no other complications reported
N. Caplin (2020) [39]	AKI	percutaneous	leakage (23%) poor flow (5%)	no infectious complications reported	bleeding (15%)
Q. Soomro (2021) [40]	AKI	percutaneous	leakage (13.16%)	no infectious complications reported	no other complications reported
S. Cho (2017) [41]	AKI	percutaneous	leakage (8%) mechanical obstruction (4%)	no infectious complications reported	no other complications reported
T. Panaput (2021) [42]	AKI	percutaneous	no mechanical complications reported	catheter infection (1.6%)	no other complications reported
H. Ye (2019) [43]	CKD	open	abdominal wall complications (0.07%)	peritonitis (0.01%)	no other complications reported
E. Wojtaszek (2019) [44]	CKD	open	leakage (11%)	peritonitis (34%)	bleeding (9%)
W. Parapiboon (2022) [45]	CKD	percutaneous	pericatheter leakage (5%)	no infectious complications reported	hemoperitoneum (5%)
H. Jin (2016) [46]	CKD	laparoscopic	catheter malposition (3.1%)	peritonitis (2.1%)	no other complications reported
M. Koch (2012) [47]	CKD	laparoscopic	no mechanical complications reported	peritonitis (1.5%)	no other complications reported

Ma et al. reported that the majority of patients in planned-start peritoneal dialysis had their catheter placed during open surgery compared to the Seldinger technique and modified Seldinger technique [48]. Moreover, their findings indicated a significant reduction in short-term catheter-related complications with the percutaneous method [48]. Similar results were reported by Hayat et al. [49].

#### 3.1.3. Initiation of Treatment and Aftercare in USPD

The 2016 update of ISPD Peritonitis Recommendations stated that systemic prophylactic antibiotics (such as cephalosporins or ciprofloxacin in cases of penicillin allergy) should be immediately administered prior to catheter insertion [50]. Additionally, topical antibiotics (such as mupirocin or gentamicin cream) should be applied daily to the catheter exit site. Alternatively, not dressing the exit site of the Tenckhoff catheter was suggested as an acceptable approach to avoid dermatitis and itching [51].

In the planned PD, the standard practice is to begin dialysis two weeks after catheter implantation [52]. USPD may be defined as starting dialysis at any point before this period, which frequently amounts to one week. However, for acute indications, such as urgent metabolic states or severe organ failure, dialysis is started on the day of catheter placement, immediately following the surgery. Initiating dialysis up to 72 h post-op may be referred to as “truly urgent” USPD [53]. The starting volume for the first few days is usually under 1 L and is gradually increased to 2 L, or other tolerable maximum volumes [43,54].

In end-stage renal disease patients, USPD should be started with low intraperitoneal volume in the supine position, and subsequently titrated to full volume if necessary [55]. In certain cases, full-volume or high-volume PD may be used depending on the patient’s requirements. Parapiboon et al. observed that minimal standard PD dosage (18 L per session) and intensive dosage (36 L per session) did not significantly differ in terms of outcomes [56].

A systematic review demonstrated that the use of icodextrin was associated with improved ultrafiltration, which was reflected in fewer episodes of fluid overload. Moreover, it led to an overall reduction in glucose exposure and absorption, as well as a decreased mortality risk compared with glucose-only PD-based solutions [57].

#### 3.1.4. Dialysis Modes for Urgent Start

Peritoneal dialysis can be performed using manual techniques, such as continuous ambulatory peritoneal dialysis (CAPD), or mechanical devices like automated peritoneal dialysis (APD). APD should be readily available for all patients initiating PD, as it offers an excellent solution for individuals with fast transport characteristics, despite the higher associated costs). Nevertheless, the most significant indication for its implementation remains the patient’s choice [28,58]. In contrast to intermittent peritoneal dialysis (IPD), low-volume tidal peritoneal dialysis (TPD) is the preferred APD mode in USPD patients due to the lower incidence of catheter-related complications [59]. Interestingly, research findings indicate that APD does not provide significant advantages over CAPD in terms of important clinical outcomes for patients with ESRD undergoing planned catheter implementation [60].

European Automated Peritoneal Dialysis Outcomes Study (EAPOS) demonstrated that a high proportion of anuric patients on APD were able to achieve dialysis and ultrafiltration targets when applying various treatment regimens [61].

While there is no standard protocol for USPD; it is advised to follow a low-volume, supine, intermittent PD approach, particularly to avoid pericatheter leaks and hernias [17].

### 3.2. Peritoneal Dialysis in Management of Acute Kidney Injury

The International Society for Peritoneal Dialysis issued its latest guidelines for the use of peritoneal dialysis in acute kidney injury (AKI) in 2020, concluding that peritoneal dialysis is suitable for the treatment of AKI in all healthcare settings [62]. The guidelines also provided suggestions for access, recommending the use of flexible peritoneal catheters when available, while other catheter types may be used if needed. The insertion procedure should be performed by trained nephrologists, interventional radiologists, or surgeons. Prophylactic antibiotic therapy was also recommended, with a preference for a closed delivery system featuring a Y connection [62]. However, despite clear guidelines and encouraging outcomes, peritoneal dialysis is still less commonly employed than hemodialysis for AKI management.

According to the 2020 guidelines of the International Society for Peritoneal Dialysis in pediatric acute kidney injury, PD represents a safe and effective kidney replacement therapy for children. The guidelines even recommend its use in low-birth-weight neonates and in neonates following heart surgery, with some technical alterations made to accommodate the low weight of the newborns, such as adjusting cycle duration and avoiding the use of automated cyclers [63]. 

Research by Sutherland et al. indicated that the incidence of AKI in hospitalized children varies depending on the diagnostic criteria used (pRIFLE, AKIN, and KDIGO), ranging between 37.3% and 51.1%, with the incidence in neonates being 29.9% [64,65]. Furthermore, AKI was associated with greater mortality and longer hospital length-of-stay (LOS) in intensive care units (ICU) [64,66]. Consequently, the choice and implementation of an appropriate treatment modality seem crucial for AKI management and improved outcomes [67,68,69,70,71]. 

**Figure 2:**
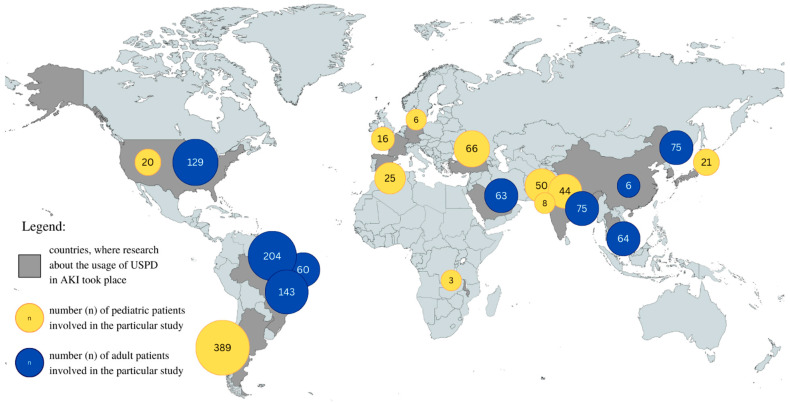
Geographical representation of USPD use in AKI in pediatric and adult populations in different countries. Graphic representation of the data in Table 2.

Our study reviewed data concerning the frequency of PD application in the AKI setting. According to the included studies, the frequency of PD administration in AKI cases ranged between 1.1% and 80% [72,73]. In a randomized controlled trial conducted by Al-Hwiesh et al., 50.4% of patients received PD [36]. Moreover, Lombardi et al. reported that 30% of units in Latin America were capable of performing PD, although only 19% actually carried out the procedure, most commonly in Brazil, Chile, Peru, and Argentina [74]. Table 2 provides a summary of the studies included in the review. Figure 2 illustrates the geographical distribution of USPD usage.

With the recent COVID-19 pandemic, healthcare systems faced numerous challenges simultaneously. AKI was a common manifestation of COVID-19, with approximately 46% of patients experiencing it and nearly 19% requiring dialysis [79]. As Sourial et al. pointed out, 35.9% of patients requiring dialysis for AKI were treated with PD during COVID-19 [77]. In turn, Caplin et al. described the initiation of a PD program during the COVID-19 pandemic, where all AKI patients were considered eligible for PD unless technical challenges rendered catheter insertion impossible or if they presented with treatment-resistant hyperkalemia requiring temporary continuous veno-venous hemofiltration (CVVH) [39].

#### 3.2.1. Indications for USPD Utilization in the Management of AKI

The timing of KRT initiation in AKI is vital, as any delay may lead to a poorer prognosis for the patient [80,81].

##### Indications for USPD in the Pediatric Population with AKI

Studies have suggested that the most common indications for PD initiation in children comprised metabolic acidosis (50.0%) and fluid overload (34.0%), followed by uremic symptoms and signs, as well as hyperkalemia [34].

Furthermore, research that did not specify the frequency of PD indication also reported other indications such as oliguria or anuria, concomitant low-cardiac-output syndrome, and increased blood urea nitrogen and creatinine with or without volume overload [33,70].

##### Indications for USPD in the Adult Population with AKI

The most common indications for PD initiation in adults involved anuria or oliguria (66%), uremic symptoms and signs (31.7–42%), high blood urea nitrogen (BUN) > 60 mg/dL (41%), acidemia (11.1–80%), refractory volume overload (25–57.3%), and hyperkalemia (25.4–52%) [35,42,78]. Additionally, high creatinine and azotemia were also considered indications; although the frequency was not provided in the studies [38,39]. Al-Hwiesh et al. observed that in the PD group, uremia and hyperkalemia were significantly more common, whereas volume overload was less common than in the continuous veno-venous hemodiafiltration (CVVHDF) group [35]. Similarly, Panaput et al. found that anuria and oliguria, as well as uremia, were more common in the PD group than in intermittent hemodialysis (IHD) and continuous renal replacement therapy (CRRT) groups [42].

#### 3.2.2. Contraindications for USPD Utilization in the Management of AKI

The ISPD guidelines for peritoneal dialysis in AKI issued in 2020 reported a few absolute contraindications, such as recent abdominal (open abdomen) surgery, abdominal compartment syndrome, and fungal peritonitis. Relative contraindications included paralytic ileus, difficulty ventilating the patient, and diaphragmatic hernia [63].

#### 3.2.3. Underlying Comorbidities in AKI

Since PD constitutes one of the modalities of KRT, it is essential to consider both the comorbidities and the initial diagnosis which may necessitate dialysis. 

In adults, the most common underlying factors leading to AKI requiring KRT included cardiovascular diseases, infectious diseases, renal diseases, respiratory diseases, and gastrointestinal diseases [35,41,42,77,78]. In addition, comorbidities in adult patients comprised diabetes mellitus, coronary artery disease, hypertension, CKD, heart failure, and liver cirrhosis [41,78].

In children with AKI treated with PD, the primary presentations often involve infectious diseases (including sepsis and malaria), renal diseases, gastrointestinal diseases, and congenital heart diseases [33,34,68].

Neonates, on the other hand, were more likely to suffer from heart disease; peripartum events; necrotizing enterocolitis; intraventricular hemorrhage; signs of fluid overload within the first 12 h, which required the use of inotropes; respiratory support; and resuscitation after delivery [67].

Moreover, peritoneal dialysis was also considered hemodynamically stable in contrast to hemodialysis; thus, it was more commonly used in hemodynamically unstable patients [42,76,81].

#### 3.2.4. Outcomes of USPD in AKI Management

PD is generally a well-tolerated method of KRT. It is also the most common KRT modality in children, particularly in low-income countries [82]. Urgent initiation of peritoneal dialysis is more commonly associated with complications than a conventional start [83]. Furthermore, outcomes may differ, depending on the patient’s state, initial diagnosis, underlying problems, and comorbidities. It is essential to consider that the mortality rate also depends on the course of the primary disease. Since patients with AKI often suffer from coexisting diseases that overlap and contribute to poorer outcomes, each patient should undergo a careful and individual evaluation process [65,84]. However, not every patient who presents with AKI requires urgent KRT. This necessity for it may reflect the severity of presentation with fluid and electrolyte imbalance, which is impossible to manage and, therefore, may be considered a worse prognostic factor in the course of the disease [65,85]. Nevertheless, our review also analyzed the PD outcomes described in the abovementioned studies, dividing them into children and adults.

##### Outcomes of USPD in Children with AKI

The most common complications described in the analyzed studies included catheter malfunction (19.6–24.0%), peritonitis (4.0–19.0%), dialysate leakage (4–11%), bleeding events (6%), hyperglycemia (2%), and catheter insertion site infection (6.1%) [32,33,34].

Moreover, patients with peritonitis received longer dialysis treatment, catheters were more likely to be implanted using an open-surgery technique, catheter malfunctions were more common, and non-cuffed catheters were more commonly used [32]. However, the majority of patients responded well to antibiotics, and catheter replacement was necessary only in 6–8% of cases [32,34]. Additionally, Coccia et al. reported that prophylactic antibiotic administration prior to PD catheter insertion was associated with a lower incidence of peritonitis, aligning with the ISPD guidelines [32,63]. Discontinuation of treatment occurred in 3.8% of patients due to peritonitis, mechanical complications, or leaks [32].

Furthermore, 1.5–6% of patients did not recover kidney function and required chronic dialysis [32,33]. In contrast, 30.3% of patients recovered their kidney function [33]. The mortality rate ranged from 2.8% to 64%, with the lowest rate associated with AKI during STEC-HUS (Shiga toxin-producing *Escherichia coli* hemolytic uremic syndrome) and the highest noted in patients in pediatric intensive care unit (PICU) [32,33].

A study by Sanchez-de-Toledo et al. demonstrated that patients who received PD within the first 24 h following heart surgery were characterized by lower mortality, shorter ICU stays, and hospitalization [75]. Kumar Sethi et al. also suggested that neonates with AKI who required PD showed significantly higher mortality, although their median length of stay in the NICU was significantly lower [67].

##### Outcomes of USPD in Adults with AKI

Panaput et al. found no significant differences in KRT duration, hospital mortality, and renal recovery between PD and other KRT modalities [42]. However, when comparing the PD group with other KRT modes, the PD group had the lowest median overall time spent in the ICU and hospital [42].

PD-related complications comprised leakage (7.3–23%), poor flow (4–5%), bleeding following catheter placement (15%), catheter-site infections, pain at inflow (2.7%), peritonitis (0–18%), hypotension (15.9%), hypomagnesemia (11.1%), and hypophosphatemia (11.1%) [35,36,37,39,41,78]. A range of 2.6–33% of patients required a change in KRT modality [39,78]. Al-Hwiesh et al. reported that all adverse effects were significantly less common in the PD group (including hypotension, infectious complications, need for catheter change, bleeding events, arrhythmias, hypoglycemia, and thrombocytopenia), except for electrolyte disturbances (hypomagnesemia and hypophosphatemia) [35]. 

According to various studies, the percentage of patients who recovered kidney function ranged from 9.3% to 60.3%, while 4.3% to 38.7% progressed to CKD, and 30.2–72% of patients died [34,35,36,37,39,41,77,78]. Randomized control trials or multicenter studies reported better outcomes [35,77]. Al-Hwiesh et al. concluded that mortality, length of stay in the ICU, infectious complications, and time to resolution of AKI were significantly lower in the PD group than in the CVVHDF group, and the percentage of patients who recovered kidney function was significantly higher [35]. Similarly, Sourial et al. found that the mortality rate was lower in the PD group compared with the extracorporeal dialysis group although LOS remained the same in both groups [77].

Sepsis was the most common cause of death [35,78]. Cho et al. observed that refractory heart failure and acute pancreatitis were associated with better survival rates, while hepatic failure, septic shock, and other causes had poorer survival rates [41]. 

In a randomized clinical trial comparing HD and PD, Ponce et al. reported that despite significant differences in fluid and electrolyte control during dialysis, the time to recovery of AKI, mortality, infectious complications, and kidney function recovery were similar in both the high-volume peritoneal dialysis group and the extended daily hemodialysis group [37]. 

**Figure 3:**
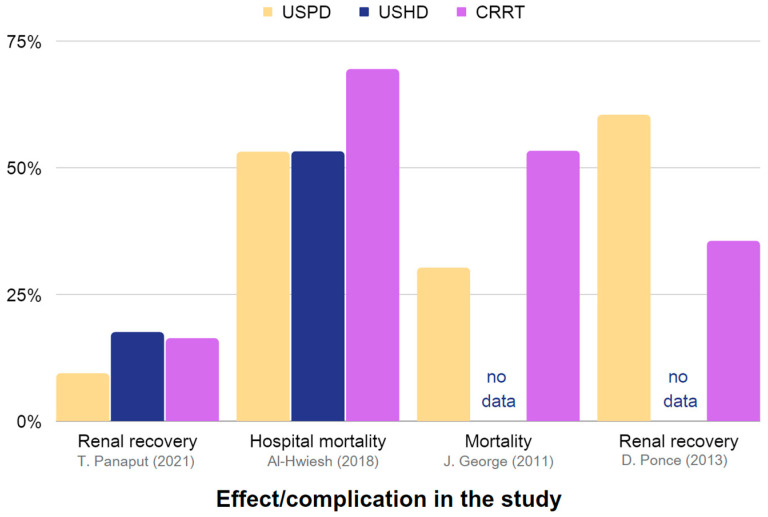
Representation of the effectiveness and associated complications of USPD, USHD, and CRRT in the management of AKI. The chart is constructed using data obtained from Table 3, which includes studies providing sufficient information [35,38,42,86].

**Figure 4:**
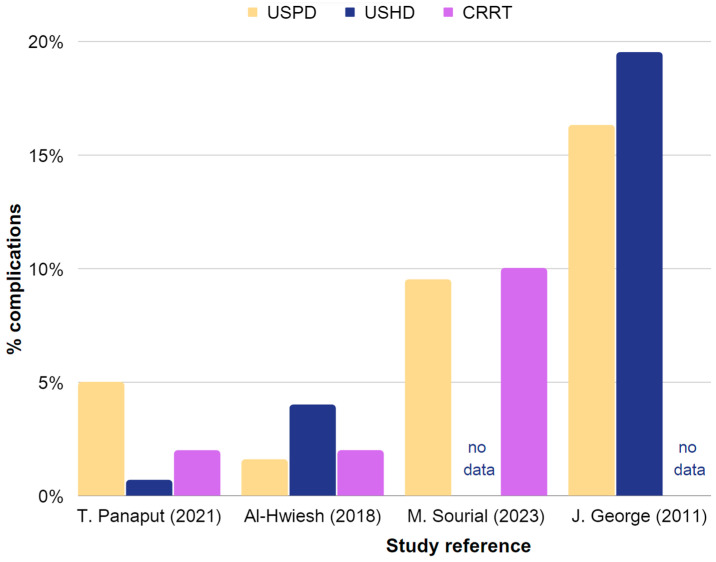
Representation of the occurrence rates of complications in USPD, USHD, and CRRT in AKI. Chart based on the data in Table 3, including studies with sufficient information. The chart is constructed using data obtained from Table 3, which includes studies providing sufficient information [35,42,77,86].

Table 3 provides a summary of the effectiveness and reported complications of USPD and USHD or CRRT in AKI. Extracted data are also presented in Figure 3 and Figure 4.

#### 3.2.5. Other Factors Influencing USPD in AKI Management

The authors of the study also examined other factors that could influence the urgent initiation of PD and its outcomes. Interestingly, Panaput et al. reported that 62.5% of PD patients were treated in regional hospitals, while IHD was used more frequently in university and provincial hospitals [42]. Furthermore, Guzzo et al. observed that PD was the predominant KRT for AKI in countries with a gross domestic product (GDP) lower than USD 35,000 per capita, whereas CRRT was more common in countries with a GDP per capita higher than USD 35,000 [88]. PD was also the most frequently used modality in larger centers with a higher volume of acute dialysis patients (>25 per year) and in hospitals where post-cardiac surgeries were common [88]. 

Coccia et al. reported that 44% of patients qualified for PD catheter insertion received antibiotic prophylaxis prior to the surgery [32]. The guidelines of the International Society for Peritoneal Dialysis recommend antibiotic administration as an optimal practice for PD in AKI [63].

Bojan et al. suggested that urine neutrophil gelatinase-associated lipocalin (uNGAL) levels could serve as a predictor for the need for dialysis and the risk of death in neonates with AKI following cardiac surgeries [70]. Moreover, Riley et al. highlighted that PD had been a standard of care for more than 20 years in the Texas Children’s Hospital Cardiovascular Intensive Care Unit (TCH CVICU) [74]. PD was initiated in children after surgical correction of complex heart disease within the first 90 days of life and those at risk for AKI or early oliguria (<1 mL/kg per h of urine output for 4 h). However, the study also indicated that KRT did not impact the recovery of kidney function [74].

In a randomized clinical trial comparing HD and PD, Ponce et al. reported that patients receiving PD were older and had higher levels of BUN and creatinine [37].

Sourial et al. reported that 47% of patients receiving PD remained in a prone position during hospitalization, compared with 70% of patients receiving extracorporeal dialysis [77]. However, the use of PD was not associated with any complications during the patients’ positioning and it was feasible to address all challenges associated with it [40,77].

Obiagwu et al. also highlighted the cost of KRT and urgent-start PD, which was found to be less expensive than HD in children [89]. Similar observations were made during the COVID-19 pandemic by Sourial et al., who concluded that PD was a viable alternative in situations of resource shortages [90]. However, they also suggested that the successful implementation of a PD program required staff training and good availability of supplies [90]. 

Table 4 provides a summary of the advantages and disadvantages of USPD in AKI, as described in this review.

### 3.3. Peritoneal Dialysis in Management of Chronic Kidney Disease

Chronic kidney disease (CKD) affects a substantial portion of the global population, with estimates suggesting that more than 800 million individuals worldwide, approximately 10% of the population, suffer from this condition [91]. CKD is particularly prevalent in older patients, women, and individuals with chronic diseases like diabetes mellitus and hypertension [92]. As a progressive disorder, CKD may ultimately lead to end-stage kidney disease (ESRD) and cardiovascular complications [93].

Despite the declining mortality rate in ESRD patients, recent reports indicate that CKD has emerged as one of the leading causes of mortality worldwide [94].

#### 3.3.1. Indications for Dialysis Treatment in CKD Management

In the 2020 report provided by KDIGO (Kidney Disease: Improving Global Outcomes), dialysis treatment was recommended for patients with CKD when one or more of the following symptoms occurred: serositis, acid-base or electrolyte abnormalities, pruritus, inability to control volume status or blood pressure, progressive deterioration in nutritional status refractory to dietary intervention, or cognitive impairment. 

CKD may also manifest with GFR ranging between 5 and 10 mL/min/1.73 m^2^ [95]. It is important to note that prognosis may differ between older and younger patients with the same GFR rate [96]. Additionally, the timing of dialysis initiation remains debatable, as studies demonstrated that early initiation of dialysis in patients with stage V CKD does not improve survival rates and clinical outcomes and may even increase the risk of death in adults [97,98,99]. Further research is needed in this area, especially regarding children under 6 years of age [100].

#### 3.3.2. Contraindications for Peritoneal Dialysis in CKD Management

The most common contraindication was abdominal scarring due to previous complex and multiple abdominal surgeries. The most crucial procedures that are included in this category are abdominal hysterectomy, prior intestinal resection, and anastomosis for intestinal obstruction [101]. Some regions also faced obstacles due to a lack of specialists trained in catheter insertion and certified nurses to perform PD [102]. Additionally, colostomy and ileostomy were usually considered contraindications for performing PD, since they entail a high risk of adhesions in the peritoneal cavity, potentially leading to mechanical problems and dialysis machine malfunctions [103].

However, it has been demonstrated that autosomal dominant polycystic kidney disease (ADPKD) should not be considered a contraindication. This group of patients showed similarities to non-diabetic patients with bilateral small kidneys [104]. Similarly, age should not be considered a contraindication for starting PD [105].

#### 3.3.3. Underlying Comorbidities in CKD

The most common underlying factors that lead to CKD and USPD involved glomerulonephritis (37–58.5%), diabetic nephropathy (9–60%), and hypertensive nephropathy (7.3–9%) [43,44,45]. Comorbid conditions included diabetes mellitus (32.3–61%), hypertension (72.9–88%), cardiac vascular disease (21.9%), and heart failure (30.2–73%) [45,46,47]. 

In patients with diabetes mellitus, special consideration is required for the impact of glucose-containing PD solutions on glucose balance and the potential disadvantages of low glucose solutions [106,107,108]. Additionally, the coexistence of obesity with CKD may increase the risk of catheter leaks, exit-site infections, a higher rate of peritonitis, and mortality [109,110]. Moreover, glucose absorption from PD solutions may potentially result in unwanted weight gain [111]. 

#### 3.3.4. Urgent vs. Conventional Start Dialysis in CKD Patients

Wojtaszek et al. observed that early mechanical complications were more frequent in urgent-start patients compared to planned-start patients (29 vs. 4%, *p* = 0.00005) [44]. Leakage was the most common issue, occurring in 11% of patients in the early phase and in 14% of cases in the late phase. Bleeding and catheter migration were the second most common complications, with a similar occurrence of 9% in the early phase after insertion. Late mechanical complications were noticed in 20% of urgent-start and 31% of the planned-start patients (*p* = 0.15). No infectious complications occurred in the first four weeks after the procedure in either group, and peritonitis rates were similar during a longer observation period, affecting 34% of patients in the urgent-start group and 33% of subjects in the planned-start group [44]. 

Chee Chin Phang et al. concluded that leakage was significantly more common in the urgent-start PD group compared with the planned-start PD group (7.6% versus 0.8%; *p* = 0.02), while there was no major difference in the occurrence of catheter malfunction (4.5% vs. 3.3%; *p* = 0.70) and catheter readjustment (1.5% vs. 2.5%; *p* = 1.00) [112]. Notably, rates of peritonitis were much higher in the urgent-start PD group (incidence risk ratio (IRR) 3.10, 95% confidence interval (CI) 1.29–7.44) and appeared noticeably earlier than in the planned-start PD group [112].

Furthermore, See et al. found that urgent-start PD patients experienced more frequent leakages (12% vs. 1%, *p* = 0.047) and catheter migration following PD initiation (12% vs. 1%, *p* = 0.047) [113]. No differences in infection rates were noted [113]. In all of the abovementioned study groups, the survival rate in the urgent- and planned-start PD was similar, which suggests that both of these methods could be successfully applied in patients with CDK.

**Figure 5:**
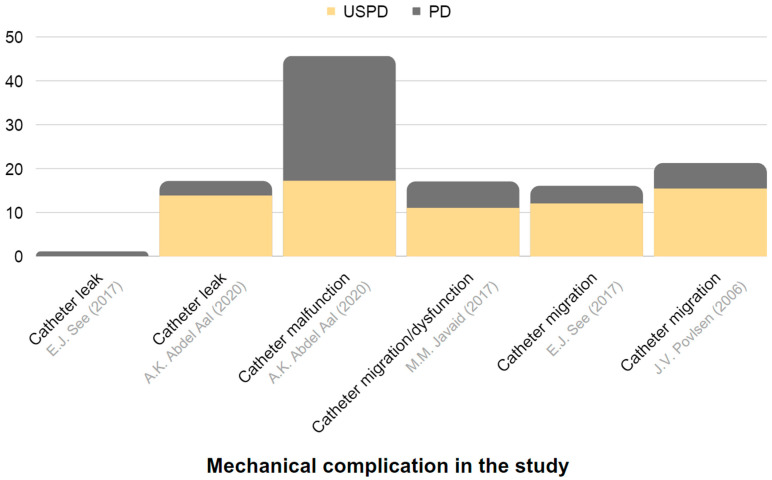
Representation of the frequency of mechanical complications in USPD and in conventional-start PD among individuals with CKD. The chart is constructed based on data derived from Table 5 [113,115,116,117].

**Figure 6:**
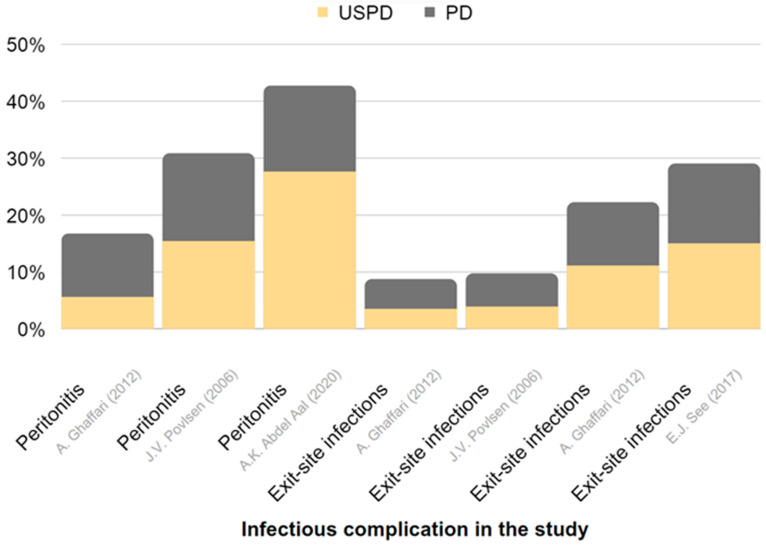
Representation of the frequency of infectious complications in USPD and in conventional-start PD in CKD. The chart is derived from data presented in Table 5 [113,114,115,116].

Table 5 and Figure 5 and Figure 6 present a comparison of urgent-start PD and planned-start PD, with USPD showing more frequent mechanical complications, such as leakages or catheter dysfunction, when compared with planned-start PD.

**Table 5:** Comparison of complications reported in USPD and in conventional-start PD in CKD.

Paper	Number of Patients	Country	USPD/PD	Mechanical Complications	Infectious Complications	Other Complications
Arshia Ghaffari (2012) [114]	18	USA	USPD	minor leaks (22.2%)	peritonitis (5.6%) exit-site infections (11.1%)	hematoma (5.6%)
	9	USA	PD	major leaks (11.1%)	peritonitis (11.1%) exit-site infections (11.1%)	no complications reported
Abdel-Aal (2020) [115]	29	USA	USPD	catheter malfunction (17.2%) catheter leak (13.8%)	peritonitis (27.6%) exit-site infections (3.5%)	hernia (10.3%)
	211	USA	PD	catheter malfunction (28.4%) catheter leak (3.3%)	peritonitis (15.1%) exit-site infections (5.2%)	hernia (4.3%) muscle hematoma/bleeding (2.8%)
Javaid et al. (2017) [116]	17	Singapore	USPD	catheter migration/dysfunction (11.0%)	no complications reported	no complications reported
	33	Singapore	PD	catheter migration/dysfunction (6.0%)	no complications reported	no complications reported
See et al. (2017) [113]	26	Australia	USPD	catheter leak (12%) catheter migration (12%)	exit-site infection (15%)	no complications reported
	78	Australia	PD	catheter leak (1%) catheter migration (4%)	exit-site infection (14%) peritonitis (3%)	no complications reported
Povlsen et al. (2006) [117]	52	Denmark	USPD	catheter leak (7.7%) catheter dysfunction (15.4%)	peritonitis (15.4%) exit-site infection (3.9%)	no complications reported
	88	Denmark	PD	catheter migration (5.8%)	peritonitis (15.4%) exit-site infection (5.8%)	no complications reported

#### 3.3.5. Outcomes of USPD in CKD Management

Hongjian et al. reported that the majority of complications following USPD occurred within a month of catheter implantation [43]. Infectious complications included peritonitis, observed in 0.01% of patients. In turn, 0.07% of patients experienced abdominal wall complications, such as hernia, hydrothorax, or pericatheter leakage.

Regarding complications resulting from the procedures, they can be categorized into mechanical (non-infectious) or infectious. Mechanical complications encompass outflow failure, leakage, hemoperitoneum, oozing from the exit site, exit site granulation, hernia, and pleuroperitoneal shunt [28]. Early complications are further classified into two subgroups: those occurring within 14 days after the procedure and those appearing after 14 days. In both subgroups, outflow failure (migration) was the most frequently reported complication.

The period free from peritonitis, mechanical complications, and exit site infection (ESI) was longer in the planned PD group, while the time to switch to hemodialysis and the survival time of patients were similar in both groups [117]. Additionally, unplanned PD was not identified as a risk factor for death, transition to HD, or complications related to therapy, although age and lower albumin concentrations were identified as predictors of negative outcomes.

Furthermore, USPD was associated with a lower requirement for vascular access procedures compared with hemodialysis, with a 2017 study showing a 1-year survival rate of 79% for this technique [118,119].

The major complications occurring in the first 30 days involved catheter tip migration and leakage [120]. Dropouts in USPD patients were primarily due to death, whereas planned PD patients were more often transferred to HD. The occurrence of complications within the first 30 days was the only risk factor for dropout. Hospitalization rates and technique survival were similar in both groups, suggesting that there were no significant differences in patients’ outcomes. The study concluded that USPD was a safe and appropriate approach [120]. 

## 4. Conclusions

Peritoneal dialysis is an effective and safe method of kidney replacement therapy. USPD provides greater cost-effectiveness and fewer infectious complications than USHD and can be considered a primary strategy in unplanned dialysis patients who require urgent care [55]. KDIGO guidelines and the International Society for Peritoneal Dialysis recommend a 2-week waiting period between creating peritoneal access and initiating dialysis [29,121]. Studies indicate that patients may undergo temporary treatment with hemodialysis if an emergency start of KRT is necessary, although USPD is also considered a safe and possible modality. ISPD guidelines for peritoneal dialysis in acute kidney injury support the application of PD as a KRT in AKI without the 2-week period [62]. 

Moreover, USPD is associated with more common mechanical complications, such as leakages, or catheter dysfunctions than planned PD. However, when implemented carefully by an experienced team, USPD indicates satisfying outcomes and should not result in more complications [122].

## 5. Future Directions

Regarding prospects, several areas of research and application of USPD appear promising in terms of enhancing its effectiveness and convenience. One crucial aspect of research involves the development of innovative dialysis solutions that optimize fluid and solute removal, while minimizing detrimental effects on the peritoneal membrane, improving ultrafiltration, reducing inflammation, and enhancing biocompatibility. 

Advancements in technology provide exciting new potential, such as the development of wearable or portable PD devices, which allow for greater patient mobility and flexibility of dialysis. Furthermore, integrating telemedicine and remote monitoring systems into PD care may provide real-time monitoring of dialysis parameters, facilitating early detection of complications and timely interventions. 

Another area of PD research involves bioengineering and tissue-engineering techniques to create bioartificial membranes with the potential to enhance biocompatibility, to reduce infection risk, and to prolong the lifespan of peritoneal access. Given the greater risk posed by USPD, it is essential to utilize the newest inventions. 

Moreover, expanding the indications for PD remains an area of ongoing research, investigating its efficacy and safety in diverse patient populations, including children, the elderly, and individuals with complex medical conditions. However, enhancing urgent PD use, both in acute kidney injury and the perioperative setting, requires refining protocols and guidelines for optimal initiation time and fluid balance management. 

Addressing the abovementioned future directions in research and application of USPD shows great potential for improving patient outcomes and quality of life in the field of kidney replacement therapy, specifically in situations where urgent intervention is required.

## Figures and Tables

**Table 2:** Usage of USPD in AKI in pediatric and adult populations in different countries and continents.

Paper	Patients Type	Number of Patients	Country	Continent
A. Riley (2015) [74]	Children	20	USA	North America
D. Hirano (2017) [69]	Children	21	Japan	Asia
I. Kaplan Bulut (2016) [33]	Children	66	Turkey	Asia
J. Sanchez-de-Toledo (2016) [75]	Children	25	Spain	Europe
M. Bojan (2014) [70]	Children	16	Paris	Europe
P. Choudhary (2021) [34]	Children	50	India	Asia
P. Coccia (2021) [32]	Children	389	Argentina	South America
R. Evans (2018) [68]	Children	3	Malawi	Africa
S. Dittrich (2000) [76]	Children	6	Berlin	Europe
S. Sethi (2022) [67]	Children	44	India	Asia
S. Nawaz (2018) [72]	Children	8	India	Asia
A. Al-Hwiesh (2018) [34]	Adult	63	Saudi Arabia	Asia
D. Gabriel (2009) [36]	Adult	60	Brazil	South America
D. Ponce (2012) [37]	Adult	204	Brazil	South America
D. Ponce (2013) [38]	Adult	143	Brazil	South America
F. Wang (2017) [71]	Adult	6	China	Asia
M. Sourial (2022) [77]	Adult	93	USA	North America
N. Caplin (2020) [39]	Adult	29	USA	North America
N. Garg (2020) [78]	Adult	75	India	Asia
Q. Soomro (2021) [40]	Adult	7	USA	North America
S. Cho (2017) [41]	Adult	75	Korea	Asia
T. Panaput (2021) [42]	Adult	64	Thailand, Indonesia	Asia

**Table 3:** Comparison of the effectiveness and complications of USPD, USHD, and CRRT in AKI.

Paper		USPD in AKI		USHD in AKI		CRRT in AKI
	Effectiveness	Complications	Effectiveness	Complications	Effectiveness	Complications
T. Panaput (2021) [42]	9.4% renal recovery	5% air embolism	17.5% renal recovery	0.7% bleeding	16.3% renal recovery	2% major arrhythmia
A. Al-Hwiesh (2018) [35]	53.1% hospital mortality	1.6% catheter infection	53.2% hospital mortality	4% major arrhythmia	69.4% hospital mortality	2% catheter malfunction
M. Sourial (2022) [77]	9 days LOS	9.5% infectious complications	16 days LOS	no data	17 days LOS	10% hypertension
D. Ponce (2013) [38]	60.3% renal recovery	no data	no data	no data	35.5% renal recovery	17.7% infectious complications
J. George (2011) [86]	30.2% mortality	16.3% infectious complications	20% renal recovery	19.5% infectious complications	53.2% mortality	no data
B. Basu (2017) [87]	9-day ICU LOS	4% hypotension	60% mortality	no data	19-day ICU LOS	no data

LOS—length-of-stay, ICU—Intensive Care Units.

**Table 4:** Advantages and disadvantages of USPD in AKI, underscoring the potential benefits of utilizing USPD in AKI management.

Advantages of USPD in AKI	Disadvantages of USPD in AKI
- Cost-Effectiveness: USPD offers a more economical option compared with other kidney replacement therapies. - Safety and Effective for Children and Neonates with Low Birth Weight. - Hemodynamic Stability: USPD is considered safe for hemodynamically unstable patients. - Well-Tolerated: Patients generally tolerate USPD well, making it a comfortable and feasible treatment approach, particularly in pediatric populations. - Effective Response to Infectious Complications: USPD-related infectious complications, such as peritonitis, show positive responses to antibiotics, contributing to successful outcomes. - Reduced Hospital/ICU Stay: USPD has been associated with shorter length of stay in hospital and intensive care units, optimizing patient recovery and overall healthcare resource utilization. - Improved Outcomes: USPD demonstrates better outcomes in terms of renal recovery and mortality rates compared with other kidney replacement modalities.	- Technical Difficulties of Catheter Insertion: The procedure for catheter insertion in USPD may pose potential technical challenges, requiring skilled medical professionals. - Staff Training and Equipment Management: Prior to catheter insertion, specialized staff training is necessary to ensure proper procedural execution. - Limited Supply Availability: USPD may face limitations in the availability of supplies, especially in certain healthcare settings or regions, which could affect its feasibility as a treatment option. - Increased Complications and Potentially Inferior Results: Urgent-start peritoneal dialysis has been associated with a higher incidence of complications and may yield less favorable outcomes when compared with conventional start methods.

## Data Availability

No new data were created or analyzed in this study. Data sharing is not applicable to this article.
